# A Dangerous School Book

**Published:** 1912-03

**Authors:** 


A Dangerous School Book.—In 1908, during Campbell's administration as Governor, the School Book Board, of which he was chairman, adopted as a text-book for Texas schools, among others, a book called "Lessons in Hygienic Physiology," by one

Walter Moore Coleman, one time principal of the Texas Normal Institute at Huntsville, Texas. The book was published by The Macmillan Company, New York, and sold to the State, on contract, at 47 cents per copy. It was put into the hands of thousands of school children. A copy of this book is before me, and has been a source of amusement; that is, it would be amusing if it were not such a serious matter, containing, as it does, so much that is false, misleading and dangerous. I quote *verbatim* from page 127:

"It has never been proved that germs will cause harm or disease to a perfectly healthy body. *Some germs change their nature or the nature of the toxins they form, according to the material upon which they live.*" (Author's italics.)

And from page 285 I quote the following:

"VENOMOUS SNAKES OF THE SOUTH named in order of virulence (based upon carefully collected cases reported to Smithsonian Institution) are as follows: 1. King Snake (Elaps). It has about seventeen red bands bordered with yellow and black. Its bite is usually fatal. 2. Rattlesnake (seldom fatal). 3. Copperhead (may kill a small animal the size of a dog). 4. Water Moccasin (never fatal). 5. Ground Rattler.

"EFFECTS OF SNAKE BITE.—Pulse fast, breathing slow, blood tubes dilated, blood becomes stored in abdominal blood-vessels, stupefaction or death results from blood being withdrawn from brain. Always two punctures, the closer together the smaller the snake.

"REMEDIES.—Ligature between wound and heart; lance wound and suck; or inject into wound three drops of 1 per cent solution of chromic acid or potassium permanganate. Take three half-grain doses of strychnine, hypodermically, twenty minutes apart."

Well, I'll stop at this, calling attention only to the learned author's injunction to "take," instead of "to give," a grain and a half of strychnine, and, "if collapse recurs," "*repeat the dose.*" It is a pity he didn't "take" it himself, before he foisted such dangerous and absurd stuff on a credulous School Book Board, and an unsuspecting thousand or so teachers and children. It is only fair to state that this book was suppressed and withdrawn when its true nature was discovered.

